# Long-Term Culture Performance of a Polyelectrolyte Complex Microcapsule Platform for Hyaline Cartilage Repair

**DOI:** 10.3390/bioengineering10040467

**Published:** 2023-04-12

**Authors:** Ehinor P. Arhebamen, Maria T. Teodoro, Amelia B. Blonka, Howard W. T. Matthew

**Affiliations:** 1 Department of Biomedical Engineering, Wayne State University, 5050 Anthony Wayne Dr., Detroit, MI 48202, USA; 2 Department of Chemical Engineering and Materials Science, Wayne State University, 5050 Anthony Wayne Dr., Detroit, MI 48202, USA

**Keywords:** articular cartilage, biomaterials, polymers, stem cells, biomechanics, osteoarthritis, engineered tissue, chondrocytes, transplantation, orthopedics, MACI

## Abstract

Articular cartilage (AC) tissue repair and regeneration remains an ongoing challenge. One component of the challenge is the limited ability to scale an engineered cartilage graft to clinically relevant sizes while maintaining uniform properties. In this paper, we report on the evaluation of our polyelectrolyte complex microcapsule (PECM) platform technology as a technique for generating cartilage-like spherical modules. Bone marrow-derived mesenchymal stem cells (bMSCs) or primary articular chondrocytes were encapsulated within PECMs composed of methacrylated hyaluronan, collagen I, and chitosan. The formation of cartilage-like tissue in the PECMs over a 90-day culture was characterized. The results showed that chondrocytes exhibited superior growth and matrix deposition compared to either chondrogenically-induced bMSCs or a mixed PECM culture containing both chondrocytes and bMSCs. The chondrocyte-generated matrix filled the PECM and produced substantial increases in capsule compressive strength. The PECM system thus appears to support intracapsular cartilage tissue formation and the capsule approach promotes efficient culture and handling of these micro tissues. Since previous studies have proven the feasibility of fusing such capsules into large tissue constructs, the results suggest that encapsulating primary chondrocytes in PECM modules may be a viable route toward achieving a functional articular cartilage graft.

## 1. Introduction

Articular cartilage (AC) tissue degeneration due to joint disease or traumatic injury results in a progressive and debilitating condition known as osteoarthritis (OA) [1,2,3,4,5]. OA afflicts approximately 20% of the U.S. population and approximately 3.8% of the global population; creating a significant socioeconomic burden, with more than USD 100 billion spent annually on treatment related expenses in the U.S. alone [1,2,3,5,6,7,8,9,10]. Regardless of the initial cause, OA is a progressive, and often irreversible condition that results in a significant loss of quality of life, especially for patients in the later stages of this disease. Current treatments range from analgesics in early-stage disease to biomaterial/cell-based repair strategies, and finally to total joint replacement in the advanced stages.

Presently, there are no effective clinical approaches that prevent the degeneration of AC tissue, and surgical interventions such as debridement or microfracture have been shown to be ineffective in preventing recurring pain for 20–30% of the patient population [4,11,12,13,14]. Additionally, the current gold standard cell-based repair approaches such as MACI^®^ face a variety of complications including implant rejection, implant material failure, scaffold migration or delamination, or fibrocartilage formation [4,15,16].

Several challenges to the successful development of a functional AC graft include: (i) mass transport limitations in tissue engineered grafts preventing scaling up to clinically relevant sizes, (ii) the presence of catabolic enzymes in the joint milieu that destroy graft extracellular matrix (ECM) components, (iii) differences in material and structural properties between healthy tissue and engineered grafts resulting in graft failure, and (iv) adverse effects of the engineered scaffold material(s) on the biological potential of seeded cells [17,18,19,20]. A considerable number of research efforts have focused on scaffolds infused with bioactive factors and cell-free or cell-laden scaffolds [8,20,21,22,23,24,25,26,27]. While some research efforts have shown promise, the major limitation to progress has been a lack of consistent biophysical properties and predictable physiological outcomes. Greater consistency would allow clinicians to better assess the performance aspects of an engineered graft to determine its safety and efficacy, potentially leading to broader adoption.

In this work, we sought to lay a foundation for addressing one major issue facing the development of a functional, tissue engineered AC graft, specifically, the ability to scale up to clinically relevant sizes. It is well-understood that the diffusion distance of O_2_ is the limiting factor in the development of clinically relevant engineered grafts. Thus, organoids and other non-vascularized large cell-based structures are characterized by diffusion-mediated mass transport limitations, resulting in cell death at the core of constructs larger than 200 µm in radius.

We previously reported a bottom–up tissue regenerative strategy that used hyaluronan (HA)-based 3D micromodules [28]. That work demonstrated that engineered constructs of clinically relevant sizes could be achieved with a modular assembly strategy whereby individual micro-modular units can be fused together to form a cell-laden tissue construct. These HA-based micromodules were bounded by a highly permeable membrane with a wall thickness of ~10 µm. Additionally, the spherical shape of the micromodules resulted in improved mass transport efficiency based on a capacity for significant flow through the inter-module void spaces in large fused-module constructs [29,30,31].

In order to optimize this design for cartilage tissue and improve the overall intra-module diffusion, we sought to develop a process for dispersing and suspending cells within an intra-module hydrogel network. This modification would accomplish two goals. First, it would facilitate a distributed cell arrangement similar to native cartilage, and second, dispersing cells would improve internal diffusion and cell survival by preventing uncontrolled cell aggregation in the early stages of module culture.

In this study, we report on an alternative strategy for generating a transplantable hyaline cartilage construct based on a polyelectrolyte complex microcapsule (PECM) composed of a crosslinkable interior gel containing methacrylated hyaluronan (HA-MA), chondroitin 4-sulfate (C4S), type I collagen, and either entrapped chondrocytes or bone marrow derived mesenchymal stem cells (bMSCs).

The capsule wall was a semi-permeable membrane composed of chitosan, HA-MA, and C4S. We assessed the effect of varying the HA-MA crosslink density on the PECM platform by evaluating the membrane structure, rate of diffusive transport, and rupture strength. Encapsulated cultures of primary chondrocytes, bMSCs, and mixed cultures of the two cell types and bMSCs were used to characterize the growth and development of cartilage like tissue as well as the development of cartilage-like physical properties over long-term (>90 days) cultures.

## 2. Materials and Methods

All materials used in this research were purchased from Sigma Chemical Co. (St. Louis, MO, USA) unless otherwise noted.

### 2.1. Modification of Hyaluronic Acid with Methacrylic Anhydride

**Figure 1:**
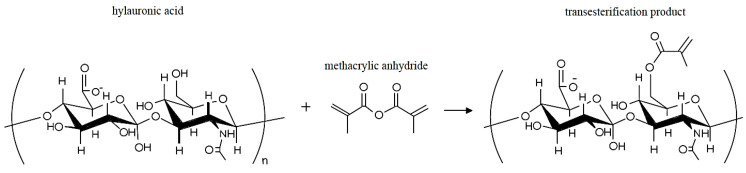
Scheme depicting the methacrylation of hyaluronic acid.

Hyaluronic acid (HA) was modified with methacrylic anhydride (MA) as previously described (Figure 1) in an established protocol [32]. Equimolar concentrations of HA (1000 kDa) and MA were reacted to obtain a 50% degree of substitution (DS) HA-MA. Generated HA-MA was purified via dialysis and freeze-dried to produce a white, fibrous product. HA-MA %DS was verified with ^1^H NMR (400 mHz Agilent). The degree of methacrylation was analyzed by calculating the ratio of the integral of MA ^1^H peaks (~6.2, 5.7 and 1.9 ppm) and HA ^1^H (~2 ppm).

### 2.2. Formation of Polyelectrolyte Complex Microcapsules

**Figure 2:**
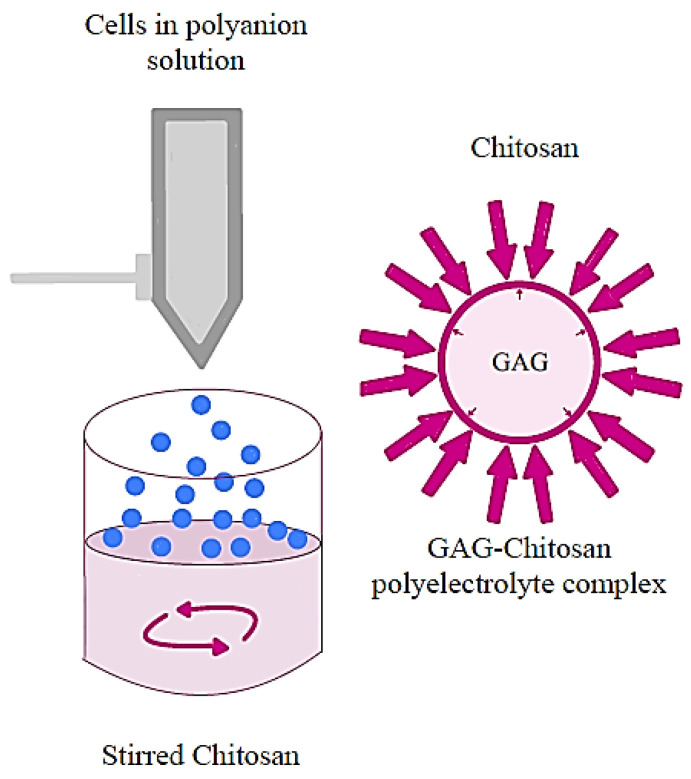
Schematic of the polyelectrolyte microcapsule formation.

The encapsulation method was previously established by Matthew et al. [33] and was used to generate hollow microcapsules with a membrane formed by the electrostatic interaction between polyanionic HA-MA and polycationic chitosan (Figure 2).

A HEPES-sorbitol buffer was prepared containing 0.04 wt% KCl, 0.05 wt% NaCl, 0.3 wt% HEPES sodium, and 3.6 wt% D-sorbitol, and pH balanced to 7.3. A 2 mL volume of polyanion solution was prepared containing 2 wt% of 50% DS HA-MA, 4 wt% chondroitin 4-sulfate (C4S), and 0.21 wt% LAP (lithium phenyl (2,4,6-trimethylbenzoyl) phosphinate) dissolved in HEPES-sorbitol buffer and autoclaved for 5 min at 121 °C. Immediately prior to capsule formation, the type 1 collagen solution (2 mg/mL in 1 mM HCl) was mixed with 10X L-DMEM in a 9:1 ratio, and 1 mL of this mixture was added to 1 mL of the polyanion solution to achieve the desired polyanion encapsulation solution concentrations. Since some cell types adhere weakly to polyelectrolyte complexes, we sought to use readily available collagen type I to provide some adhesive protein sequences in support of cell adhesion, migration, and proliferation in the early stages of culture. Collagen type I has also been used in multiple studies of articular cartilage tissue engineering where it supports early cell organization without promoting adverse effects [34]. Droplets of the polyanion solution (~0.8 mm diameter) were generated by air entrainment from a 24-gauge Teflon catheter and collected in 30 mL of stirred chitosan solution (0.6 wt%, 90% deacetylated chitosan, 3.8 wt% sorbitol, 0.12% (*v*/*v*) glacial acetic acid) containing 2–3 drops of Tween-20. Care was taken to generate uniform droplet sizes. Capsule membranes were formed almost instantaneously via ionic complexation between the oppositely charged polymers. Immediately following droplet collection and capsule membrane formation, the stirred capsule suspension was irradiated with violet light (405 nm) for 3 min. The formed capsules were then washed in a 0.9 wt% normal saline solution, followed by a wash with 0.1 wt% polygalacturonic acid (PGA) in HEPES-sorbitol buffer. Capsules were then equilibrated with Hank’s Balanced Salts solution. The PECM membrane structure and interior gel distribution were assessed using scanning electron microscopy (SEM, JEOL JSM-7600). To prepare the samples for SEM imaging, the microcapsules were first fixed in a 2.5% (*v*/*v*) glutaraldehyde solution in PBS for 24 h at 4 °C, followed by three PBS (pH 7.4) washes. Samples were then dehydrated using an ethanol series from 30% to 100%. Finally, the samples were placed in 70% ethanol and snap frozen in liquid nitrogen, freeze-dried, mounted on aluminum stubs, cracked open, and sputter coated with gold.

### 2.3. Assessing Mass Transport in Polyelectrolyte Complex Microcapsules

Diffusion of bovine serum albumin (BSA) was used to characterize mass transport through the PECM membrane and interior gel, as previously described [28]. Cell-free capsules were equilibrated with BSA and the outward diffusion of BSA from the capsules was measured. This diffusion rate was used to estimate the overall mass transfer coefficient and permeability. Briefly, microcapsules were equilibrated for 48 h at 4 °C in Hanks Balanced Salt Solution (HBSS) containing 2 mg/mL bovine serum albumin (BSA) and 1 mg/mL FITC-BSA. This allowed the BSA binding sites on the capsule wall to become fully saturated. Capsules were washed twice with HBSS and then suspended in fresh HBSS. Washed capsules were then distributed into three quartz fluorescence cuvettes (~20 in each) and the total volume of HBSS + capsules in each cuvette was brought up to 3 ml. Cuvettes were sealed and briefly mixed by inversion and the fluorescence of the external solution was measured at t = 0. All three cuvettes were placed horizontally on an orbital shaker set at 120 rpm. The outward diffusion of BSA was evaluated by measuring the fluorescence of the external solution every 15 min over 3 h. At every time point, the capsules were allowed to settle to the bottom of the cuvettes, prior to performing the fluorescence measurements (FITC, Ex/Em 495/520 nm). The BSA concentration inside the solution was then calculated using a BSA standard curve covering 0–50 µg/mL. The following unsteady state and instantaneous mass balances were used to model the diffusion of BSA and hence the mass transfer coefficient (*K*) of the capsules:

(1) $$V\frac{dC}{dt}=KA\left(C_{c}-C\right)$$

(2) $$NC_{c}V_{c}+VC=NC_{1}V_{c}+VC_{0}=M$$

where *M* is the total mass of the solute present in the cuvette; V, $V_{c},$ and N are the volume of the external solution, the volume of a capsule, and the number of capsules, respectively; C, $C_{0}$, $C_{c}$, and $C_{1}$ are the concentration of the solute in the external solution at time *t*, the initial extracapsular concentration, the concentration of solute inside the capsules at time *t*, and the initial intracapsular concentration, respectively. Solving Equations (1) and (2) produces Equation (3):

(3) $$\ln\left(Q\right)=\left(\frac{KA\left(V+NV_{c}\right)}{NVV_{c}}\right)t$$

where *Q* is a dimensionless, concentration-dependent parameter defined as:

(4) $$Q=\frac{M-\left(V+NV_{c}\right)C_{0}}{M-\left(V+NV_{c}\right)C}$$

The value of *K* was derived from the slope of the linear region of the *ln*(*Q*) vs. time plot. Finally, the permeability, *P*, of each capsule was calculated with Equation (5), where *δ* is the average capsule wall thickness.

(5) $$K=\frac{P}{\delta }$$

### 2.4. Assessing Capsule Rupture Strength

**Figure 3:**
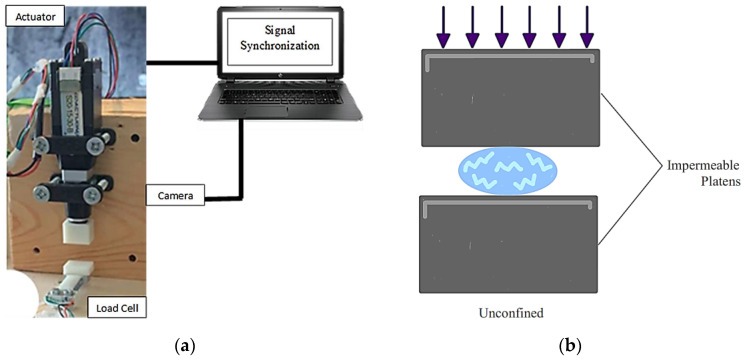
(**a**) Compression testing diagram. (**b**) Diagram of unconfined compression.

The PECM compressive rupture strength was measured at a constant compression rate (10 mm/s) under unconfined compression conditions. Briefly, individual PECMs were placed onto a 100 g load cell and then a stepper motor-driven actuator was utilized to compress the PECMs until rupture. The load cell was calibrated, and the video frame data were synchronized with the load cell output. Three pieces of data were collected: load cell voltage, video frame rate data, and digital video. These datasets were synchronized to visualize and determine the exact moment of capsule rupture and the load being applied (Figure 3). Values from a minimum of six capsules were used to determine the standard deviation.

### 2.5. Isolation and Culture of Chondrocytes and bMSCs

Tissue harvesting was conducted according to the guidelines and recommendations set in the Guide for the Care and Use of Laboratory Animals from the National Institutes of Health. All experimental protocols were reviewed and approved by the Institutional Animal Care and Use Committee at Wayne State University. Chondrocytes and bMSCs were isolated from the femurs of euthanized, 6-month-old, Sprague Dawley (SD) rats, weighing 200–250 g, according to standard protocols. In brief, the femur was exposed by clearing the surrounding soft tissue, and the bone were removed by cutting the connective tissue on either end. Sections of cartilage were taken from the femoral head with a scalpel and placed into 0.20 wt% collagenase II in chondrocyte basal medium overnight under standard cell culture conditions. Once the tissue was digested, chondrocytes were pelleted by centrifugation at 300× *g* for 5 min, then re-suspended in fresh chondrocyte maintenance medium composed of 50:50 L-DMEM/Ham’s F-12 supplemented with 10% FBS, 1% ITS, 50 µg/mL L-ascorbic acid-2-phosphate, 100 nM dexamethasone, 10 ng/mL TGF-β3, 50 µg/mL gentamycin, and 2.5 µg/mL amphotericin B. The cells were plated on standard tissue culture plastic at a density of 20,000 cells/cm^2^. bMSCs were isolated using an established procedure [35]. To isolate the bMSCs, the femur was cut at both ends and flushed three times with an 18-gauge needle and syringe delivering 10 mL of bMSC expansion medium (L-DMEM supplemented with 10% FBS, 50 µg/mL gentamycin and 2.5 µg/mL amphotericin B). Once isolated, the bone marrow cells were pelleted at 300 g for 5 min then washed with D-PBS (pH 7.3). This process was repeated 3 times to maximize the removal of erythrocytes. Finally, isolated bMSCs were re-suspended in fresh medium and plated on tissue culture plastic at a seeding density of 20,000 cells/cm^2^. Residual erythrocytes were removed during subsequent medium changes. Chondrocytes and bMSCs were allowed to proliferate, then sub-cultured at 80% confluence. Cells were allowed to reach the fourth passage prior to testing.

### 2.6. 28-Day Encapsulated Culture of Chondrocytes and bMSC

**Figure 4:**
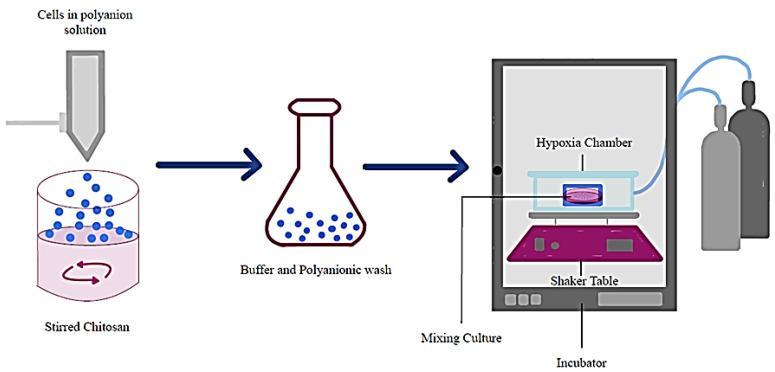
Diagram of the encapsulation culture process.

Chondrocytes were detached from the cell culture plates by treatment with 0.20 wt% collagenase II in the chondrocyte basal medium. Chondrocytes were incubated in this solution overnight under standard cell culture conditions. Once detached, the cells were pelleted by centrifugation (300× *g* for 5 min). bMSCs were detached with a 0.25 wt% trypsin-EDTA solution for 5 min. The trypsin was neutralized with bMSC basal media and the bMSCs were pelleted by centrifugation (300× *g* for 5 min). For encapsulation, chondrocytes or bMSCs were suspended at a concentration of 10 million cells/mL in 1 mL of the polyanion solution (1 wt% 50% DS HA-MA, 2 wt% C4S, 1 mg/mL collagen I, 0.1 wt% LAP), then loaded into the droplet forming apparatus. Droplets of the cell suspension (~0.8 mm diameter) were generated and collected into 30 mL of stirred chitosan solution to form cell-laden PECMs, which were then photo-crosslinked, washed, and stabilized as described above. After stabilization, the capsules were equilibrated with culture medium for ~1 h to allow for the out-diffusion of excess interior chondroitin 4-sulfate, and were then transferred to fresh medium for ongoing culture. For the chondrocyte cultures, cell-laden capsules were cultured in chondrocyte maintenance medium. For the bMSC cultures, the cell-laden capsules were cultured in chondrogenic induction medium for 7 days, then cultured in chondrocyte maintenance medium (described above) for the remainder of the study. The chondrogenic induction medium was composed of 50:50 L-DMEM/F-12 supplemented with 2% FBS, 10% ITS, 50 µg/mL L-ascorbic acid-2-phosphate, 100 nM dexamethasone, 10 ng/mL TGF-β1, 50 µg/mL gentamycin, and 2.5 µg/mL amphotericin B. Approximately 30 cell-laden PECMs were then placed into each well on 96-well tissue culture plates in a fluid volume of 200 µL per well. The plates were placed into an incubator under hypoxic conditions (2% O_2_, 5% CO_2_, balance N_2_) with mixing via a rotary shaker table operating at 60 rpm (Figure 4). Culture media were changed every 3 days.

### 2.7. Assessing the Effect of the PECM Culture on Chondrocytes and bMSCs

Changes to the physiological condition and material properties of the system were assessed every 7 days over a 28-day period. The PECM membrane integrity was assessed by measuring the rupture strength at a constant loading rate (10 mm/s). Capsules were imaged using brightfield microscopy, live/dead fluorescence microscopy, and SEM. Preparation for SEM was conducted according to the standard protocols [36]. Briefly, microcapsules were fixed in 3% glutaraldehyde in 0.1 M cacodylate buffer overnight at 4 °C. Microcapsules were then washed 3× with PBS buffered to pH 7.3, then dehydrated through an ethanol series (30–100%). Dehydrated microcapsules were then placed in 70% ethanol and snap frozen in liquid nitrogen. Then, the microcapsules were freeze-dried. Once dried, the capsule samples were cut in half with a scalpel, mounted onto SEM stubs, and sputter coated with gold. The samples were then imaged according to the standard SEM usage protocols.

### 2.8. 90-Day Encapsulated Cell Cultures

Under aseptic conditions, the bMSCs and chondrocytes were separately prepared and encapsulated as described above for the 28-day cultures. For the mixed-cells culture condition, bMSCs and chondrocytes were prepared by first detaching and collecting the individual cell types as described above. Suspensions of five million cells of each cell type were mixed in a 1:1 ratio and then pelleted. The mixed bMSC/chondrocyte cell pellet was then resuspended in the polyanion solution at a density of 10 million mixed cells/mL and capsules were generated as described above. The chondrocytes-only and bMSC-only capsules were cultured in their respective media as described above. For the mixed culture, cell-laden capsules were cultured in chondrocyte maintenance medium for the entirety of the study. Cell-laden PECMs were cultured for 90 days under hypoxic conditions with mixing as described above.

### 2.9. Assessing the 90-Day 3-Cell Condition Encapsulation Culture

Over the course of the 90-day culture, capsule samples were collected at 30-day intervals and the capsule-cultured cells were assessed with regard to their levels of DNA, sulfated GAGs, and collagen. Capsule cultures were imaged using brightfield and confocal microscopy (calcein AM/ethidium homodimer). The capsule samples were also processed for SEM imaging as described above. For the following biochemical assays, approximately 30 capsules from each culture well were crushed and digested with 100 μL of the appropriate cell lysis or digestion solution. Capsule fragments were removed by centrifugation prior to performing assays on the digest. The DNA content was measured using a HOECHST 33258 assay [28]. The total collagen content was measured after acid hydrolysis using a hydroxyproline colorimetric assay [37]. Sulfated GAGs were measured using the dimethylmethylene blue (DMMB) colorimetric assay [38]. The PECM membrane integrity was assessed by measuring the rupture strength of individual capsules at a constant compression rate (10 mm/s). All quantitative measurements were performed with a sample size of n = 6 for statistical purposes.

## 3. Results

### 3.1. Material Characteristics of the Polyelectrolyte Complex Microcapsule

The methacrylation of hyaluronic acid grafts methacrylic acid onto the 6-carbon of the acetylated glucosamine moiety of the repeating unit. This change has the potential to alter the hydrogen bonding, and ultimately, the microarchitecture of the polyelectrolyte complex formed with chitosan. We therefore sought to evaluate the morphology of the capsules and the micro-architecture of the PECM membrane.

#### Polyelectrolyte Complex Microcapsule Morphology and Permeability

**Figure 5:**
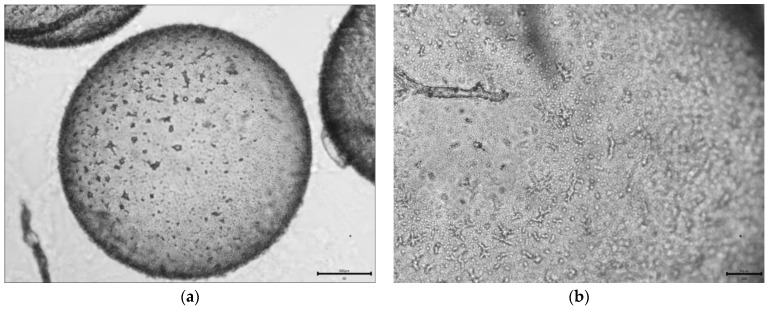

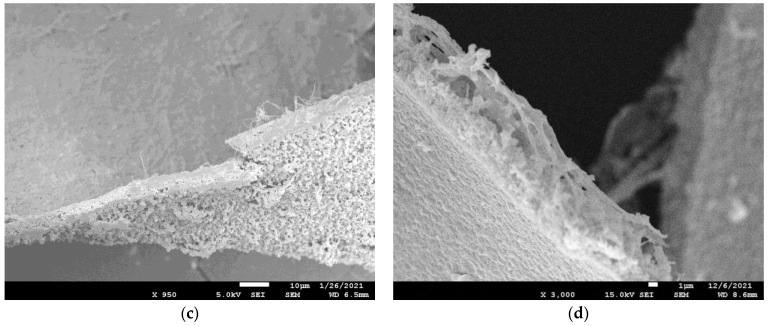
Images of a formed PECM. (**a**,**b**) Brightfield images of PECM. (**c**,**d**) SEM images of the cross-sectional view of the capsule wall showing the internal and external membrane surfaces.

Brightfield imaging (Figure 5a) showed a spherical microcapsule with a well-defined membrane. Polyanion droplets 0.8 mm in diameter produced microcapsules with a diameter of 1.0 ± 0.15 mm. Figure 5b shows a higher magnification view of the PECM membrane. The spots appear to be features developed during membrane formation. SEM images (Figure 5c,d) showed that the capsules were hollow with 10–12 μm thick membranes having a nanofibrous or nanoporous architecture. The interior surface of the capsules appeared to have a fibrous quality, likely due to the assembly of collagen fibers on this surface during and after membrane formation, while the external aspect of the membrane surface had a more granular appearance.

**Figure 6:**
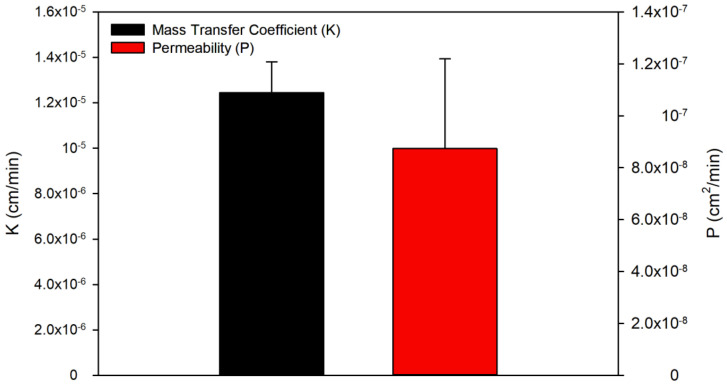
Comparison of the mass transfer coefficient (K) and capsule wall permeability (P). Error bars indicate the standard deviation.

Since the PECM membrane and any interior gel represent a diffusion barrier to nutrients, cell signaling molecules, and cell products, we sought to characterize the magnitude of the barrier by determining the system permeability to BSA. The overall mass transfer coefficient for BSA diffusion from the capsules was used to estimate the permeability of the PECM membrane to BSA, with the permeability serving as a surrogate for the mass diffusivity of BSA within the capsule membrane. The mass diffusivity coefficient (P) of BSA in an aqueous solution at pH 7 was reported to be 9.89 × 10^−7^ cm^2^/s [39] or 5.93 × 10^−5^ cm^2^/min. In comparison, the capsule permeability to BSA was 8.9 × 10^−8^ cm^2^/min (Figure 6). These values indicate a >600-fold decrease in BSA diffusivity within the PECM membrane compared to the free solution.

### 3.2. Morphology of Chondrocytes and bMSCs over 28-Day Encapsulated Culture

**Figure 7:**
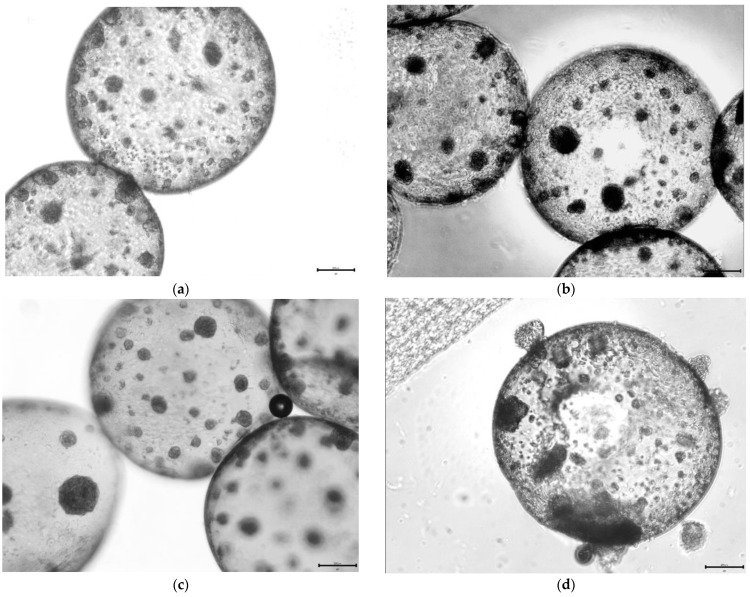

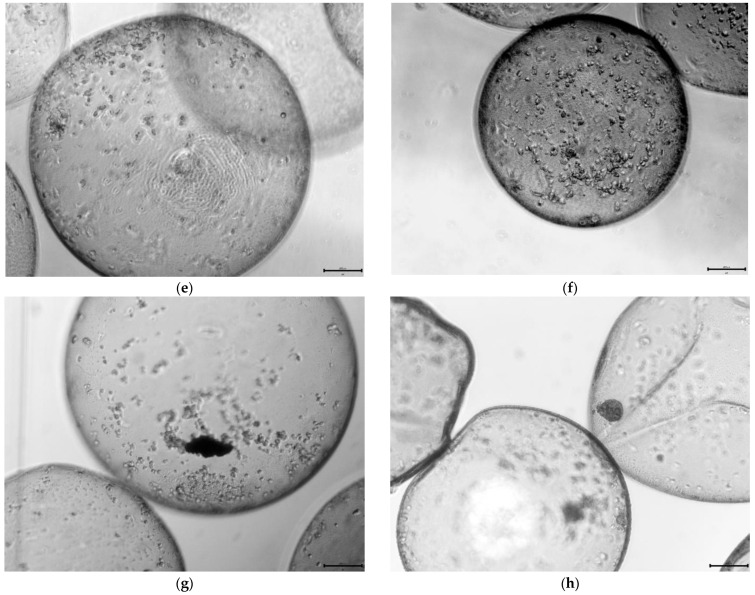
Brightfield images of 28-day chondrocyte and bMSC encapsulated cultures. (**a**) Chondrocytes day 7; (**b**) chondrocytes day 14; (**c**) chondrocytes day 21; (**d**) chondrocytes day 28; (**e**) bMSC day 7; (**f**) bMSC day 14; (**g**) bMSC day 21; (**h**) bMSC day 28.

**Figure 8:**
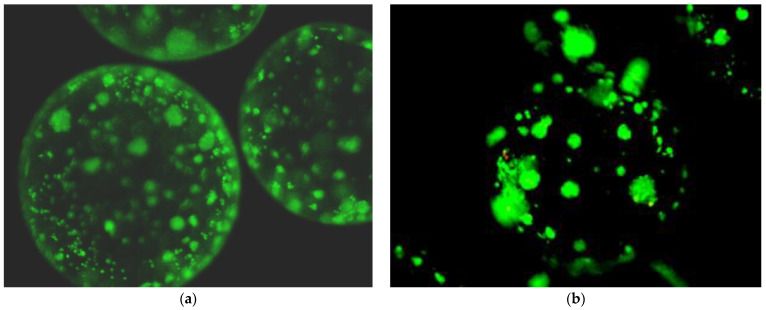

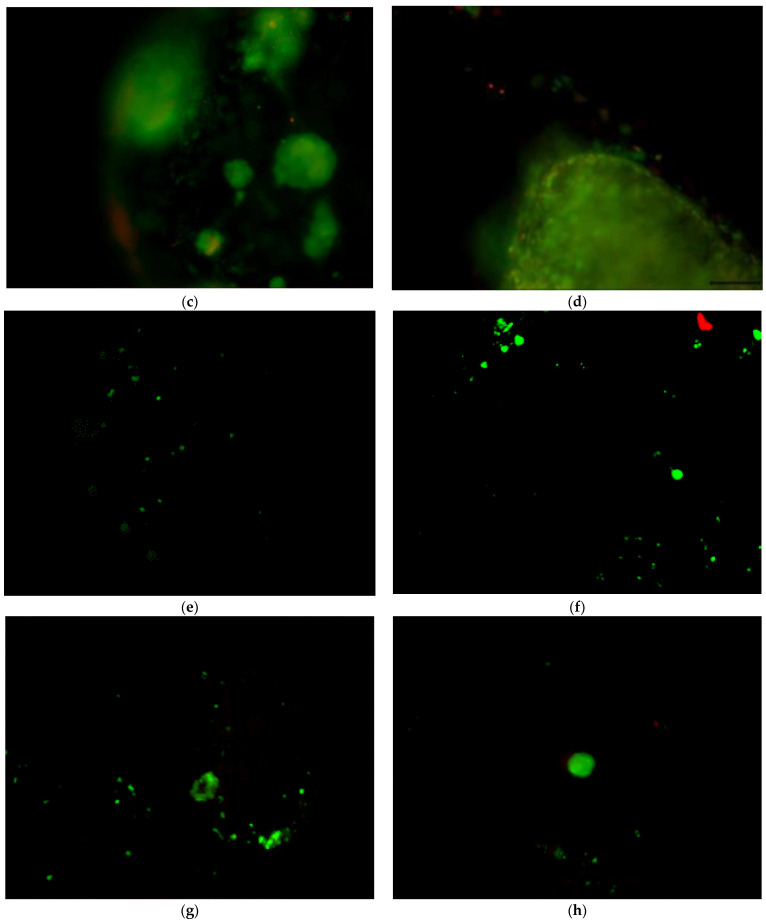
Calcein AM/ethidium homodimer (live/dead) fluorescence images of the 28-day chondrocyte and bMSC encapsulated cultures. (**a**) Chondrocytes day 7; (**b**) chondrocytes day 14; (**c**) chondrocytes day 21; (**d**) chondrocytes day 28; (**e**) bMSC day 7; (**f**) bMSC day 14; (**g**) bMSC day 21; (**h**) bMSC day 28. All images were captured with a × objective.

**Figure 9:**
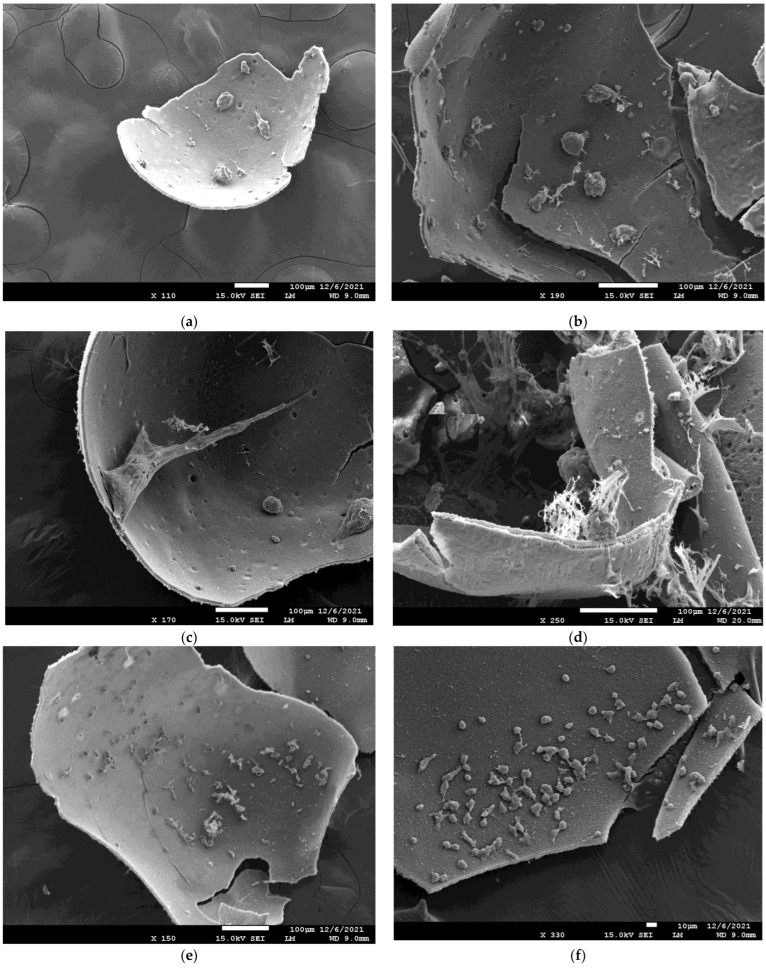

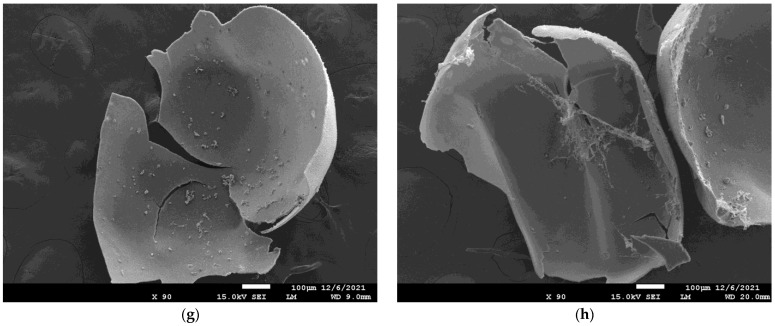
The SEM images of the 28-day chondrocyte and bMSC encapsulated cultures. (**a**) Chondrocytes day 7; (**b**) chondrocytes day 14; (**c**) chondrocytes day 21; (**d**) chondrocytes day 28; (**e**) bMSC day 7; (**f**) bMSC day 14; (**g**) bMSC day 21; (**h**) bMSC day 28.

To characterize the morphology of encapsulated chondrocytes and bMSCs, we conducted brightfield and fluorescent (live/dead) imaging, along with SEM imaging of the encapsulated cultures at intervals over a 28-day culture. In the chondrocyte-seeded capsules (Figure 7a–d), brightfield imaging showed chondrocytes forming small cell aggregates early by day 7. Larger aggregates were seen to be adhered to the interior membrane wall of the PECM. By day 21, chondrocyte aggregates were observed to be merging to form larger aggregates. In the bMSC condition (Figure 7e–h), significant aggregation was not observed until day 21. Additionally, the density of cells in this condition appeared to decrease over time. In the live/dead fluorescence images, aggregates could be seen from days 7 to 28 in the chondrocyte condition (Figure 8a–d). However, in the bMSC condition, only a few small aggregates were observed between days 21 and 28 (Figure 8e–h). SEM images of the chondrocyte condition (Figure 9c) showed that large cell aggregates and a fibrous matrix had developed inside the PECM. In the bMSC condition, no aggregation and very little matrix accumulation was observed until day 28 (Figure 9d).

### 3.3. Changes in PECM Rupture Strength during Culture

**Figure 10:**
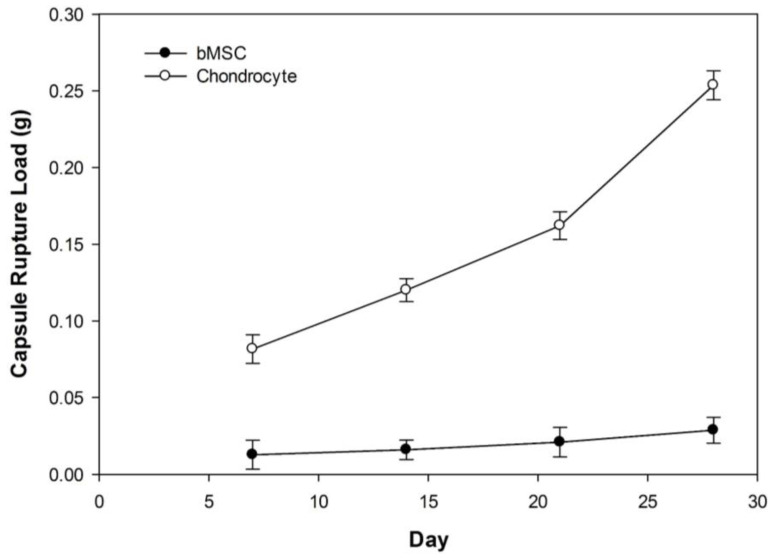
The 28-day encapsulation culture PECM rupture strength analysis. Error bars indicate standard deviation.

Since PECM materials are enzymatically degradable, and the cell deposition of extracellular matrix components was expected, the compressive rupture strength of individual capsules was measured to track changes as a function of time in culture. A plot of capsule rupture strength (Figure 10) showed that while both cell types produced a steady increase in compressive rupture load over the culture time, the chondrocyte PECM produced about a 10-fold greater increase in the rupture strength than the bMSC PECMs.

### 3.4. 90-Day Encapsulation Cultures

**Figure 11:**
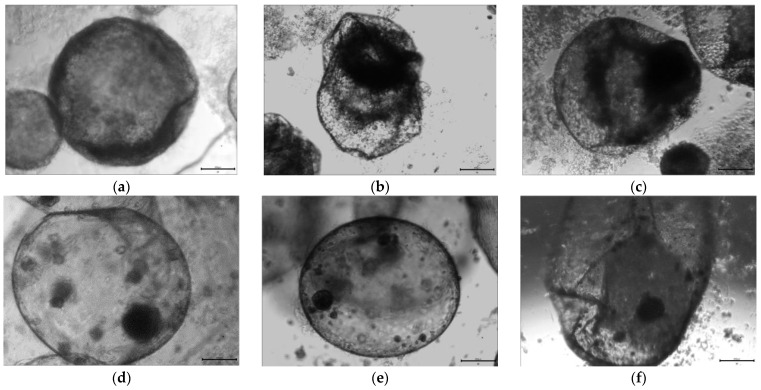

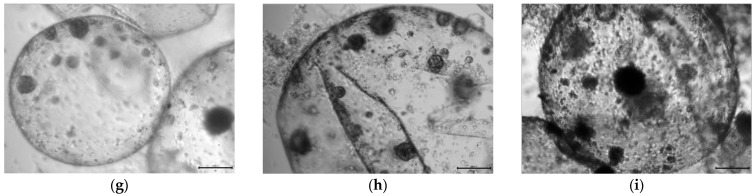
Brightfield microscopy images of the 90-day encapsulation culture. (**a**–**c**) Chondrocyte days 30, 60, and 90; (**d**–**f**) bMSC days 30, 60 and 90; (**g**–**i**) mixed days 30, 60, and 90.

In this segment, we characterized the effects of long-term (90-day) culture on the properties of PECMs containing chondrocytes, bMSCs, and a 50:50 mixture of the two cell types. We also sought to understand the effects of including type I collagen as an interior gel component. Finally, we sought to determine whether co-culture with chondrocytes could induce bMSC differentiation into a chondrocyte-like phenotype in the absence of TGF-β1. Brightfield images of the chondrocyte PECM (Figure 11a–c) showed high aggregate density and cells distributed throughout the capsule at day 30. Between day 60 and 90, increased capsule deformation and contraction was observed together with cell escape and external growth of cell masses on both capsules and the culture dish. Images of the bMSC condition (Figure 11d–f) showed smaller, denser aggregates forming at day 30, but no capsule deformation was observed until ~day 90. Images of the mixed, bMSC/chondrocyte condition (Figure 11g–i) showed cell aggregates forming at day 30, and some capsule deformation occurred by day 60. However, beyond day 60, the system seemed to stabilize, and no other significant changes were observed (Figure 11g–i).

**Figure 12:**
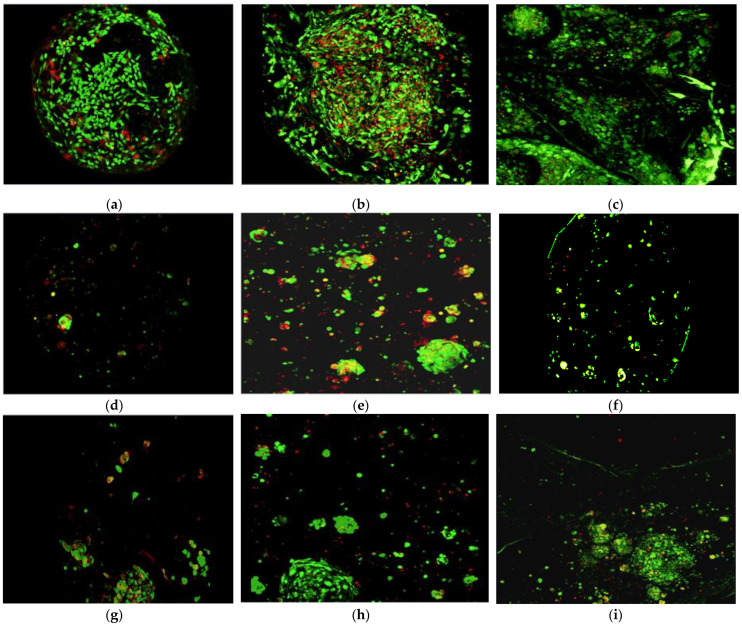
Confocal live/dead fluorescence images of the 90-day encapsulation culture. (**a**–**c**) Chondrocyte days 30, 60, and 90; (**d**–**f**) bMSC days 30, 60, and 90; (**g**–**i**) mixed days 30, 60, and 90.

**Figure 13:**
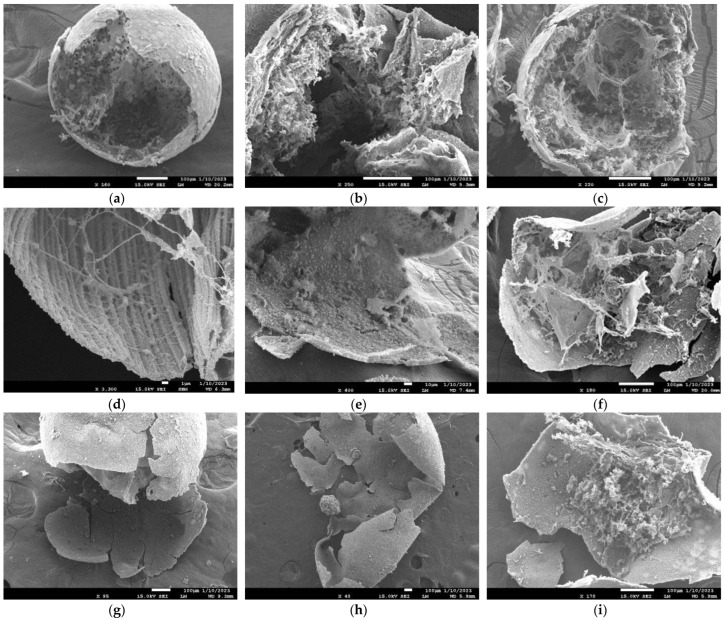
Scanning electron microscopy images of the 90-day encapsulation culture. (**a**–**c**) Chondrocyte days 30, 60, and 90; (**d**–**f**) bMSC days 30, 60, and 90; (**g**–**i**) mixed days 30, 60, and 90.

Live/dead fluorescence images (Figure 12) showed that most of the cells in every condition tended to aggregate close to the capsule wall. Notably in the chondrocyte culture, cell aggregates tended to increase in volume and fused into a single cell mass that exhibited significant folding in the later stages of culture (Figure 12a–c). The bMSC culture exhibited little cell aggregation until day 60, and there was some decline in the size of the aggregates toward the end of the culture. The SEM images (Figure 13) showed that a dense, space filling matrix was formed within the chondrocyte condition by day 30 and that the morphology was maintained through to day 90. While a fibrous extracellular matrix (ECM) was also seen in the bMSC and mixed culture capsules, it was much less extensive in the bMSC condition and was only noted at day 90 of the mixed cell condition. These results clearly demonstrate the superiority of the chondrocyte capsule cultures over the chondrocytically induced bMSCs, particularly with regard to the generation of a space-filling interior ECM.

**Figure 14:**
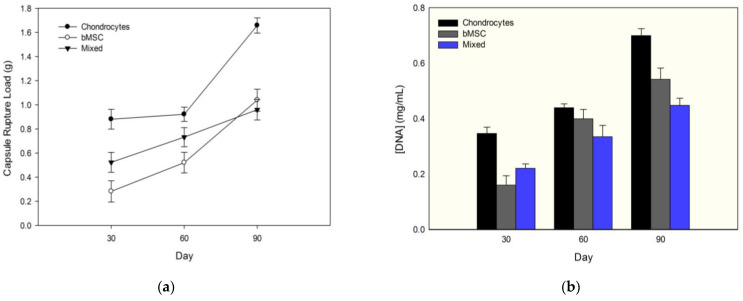
Quantitative measurements of the changes over the 90-day encapsulation culture to (**a**) the PECM rupture strength and (**b**) cellular DNA content. Error bars indicate the standard deviation.

Measurements of the capsule compressive rupture strength (Figure 14a) showed that over the course of 90 days, all three cell cultures showed continuous increases in strength. Chondrocyte capsule cultures demonstrated the highest strength values from days 30 to 90. The bMSC capsules showed the lowest early rates of strength increase, but demonstrated a faster increase rate over the last 30 days and ultimately reached levels comparable to the mixed capsule culture at day 90.

The DNA quantification results (Figure 14b) also showed continuous increases in the DNA content in all three cell conditions, with the highest growth rates seen early in the chondrocyte culture. Interestingly, the mixed cell capsules showed the lowest DNA content later in the culture, suggesting that there was no proliferation boost provided by the presence of bMSCs.

**Figure 15:**
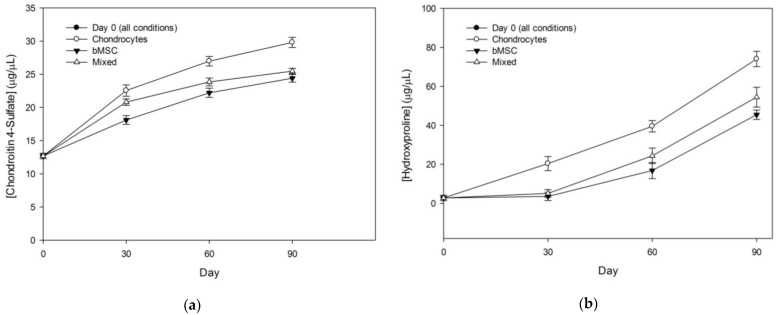
Quantitative measurements of the cartilage matrix components deposited by cells during the 90-day encapsulation culture. (**a**) Sulfated GAGs expressed as chondroitin sulfate; (**b**) collagen content expressed as hydroxyproline. Error bars indicate standard deviation. Note that day 0 values reflect the inclusion of chondroitin 4-sulfate and collagen I in the initial encapsulation solutions.

Since cartilage properties are mostly dependent on the composition and architecture of the ECM, the deposition of the ECM components was assessed by quantifying the levels of sulfated GAGs (as chondroitin sulfate) and collagen (as hydroxyproline) incorporated into the capsules. Deposition of sGAG (Figure 15a) increased with time in all conditions but tended to approach a plateau by day 90. In contrast, the collagen deposition showed an increasing rate of accumulation in all conditions over the culture period (Figure 15b). In both cases, chondrocytes showed the highest levels of deposition at all time points with the mixed culture performing at an intermediate level.

## 4. Discussion

Modular assembly schemes are an efficient method of building large complex systems, as seen in both nature and man-made constructions. In nature, modularity promotes scalability in the sense that individual cells possess the ability to self-organize on a scale that is limited by the diffusion distance of oxygen and small molecule nutrients, typically a few hundred microns. Self-organized modules of such size can then be precisely assembled in a fashion that generates tissues with a high degree of spatial organization and specific function. The liver and kidney are two organs in the human body that are modular constructs whose specific structures define their functions. Articular cartilage, while simpler in architecture, has a structure that may be particularly amenable to a modular construction approach.

As stated earlier, most efforts at cartilage engineering have focused on either infusing bulk scaffolds with bioactive factors or attempting to recreate the 3D geometry that chondrocytes experience in their native environments [8,20,21,22,23,24,25,26,27]. In general, we sought to address one major issue facing the development of a functional, articular cartilage graft, specifically, designing a graft that can be scaled up to clinically relevant sizes. It is well-understood that the diffusion distance of oxygen is a major limiting factor in the development of clinically relevant, engineered grafts [40]. Using a modular approach based on polyelectrolyte microcapsules allows tissue designers to create capsule-based tissue modules that can be matured in vitro under optimal, non-diffusion limited conditions. After module maturation, the polyelectrolyte capsule membrane allows for a number of ionic or covalent chemistries by which modules can be fused into either micro-vascularized [28] or, in the case of cartilage, avascular bulk tissue constructs.

During this study, hyaluronic acid was methacrylated with the intention of generating a photocrosslinked interior gel capable of dispersing the encapsulated cells and preventing early cell settling and aggregation. However, at the final concentrations employed (1 wt% HA-MA), the encapsulated cells did not appear to be suspended, and no interior gel could be confirmed. Furthermore, the SEM images showed settled and aggregated cells within hollow capsules. We postulate that HA incorporation into the PECM membrane during membrane formation likely depleted the interior solution to the extent that the formation of a continuous gel was impossible.

In the long-term, 90-day cultures, we found that the primary chondrocytes significantly outperformed bMSCs with regard to generating robust, matrix-filled capsules with increased load-bearing capacity. The SEM imaging showed that chondrocytically-induced bMSCs produced a significant amount of interior ECM only after day 60, while the chondrocyte culture produced notable accumulations of ECM by day 30. Interestingly, the mixed chondrocyte-bMSC capsules appeared to outperform the bMSCs in growth at day 30 but then lagged in cell growth during the later stages of the extended culture. This observation suggests that the close proximity of chondrocytes and bMSCs was not sufficient to induce the robust differentiation of bMSCs to a highly proliferative chondrocytic phenotype.

It was also observed that the sGAG deposition rates decreased over time for all cell conditions while the collagen deposition rates increased with time. We postulate that the lack of significant mechanical stimulation during culture might be the reason for the reduction in the sGAG accumulation rate over time. Proteoglycans rich in sGAG such as aggrecan are primarily responsible for the compressive strength of cartilage tissue [38] and their production is upregulated in response to cyclic compressive stresses, which were minimal in our cultures. An alternate explanation for these observations suggests that increasing collagen deposition rates might be correlated with a trend toward chondrocyte hypertrophy, possibly leading toward ossification of the culture. Analysis of this possibility would require the direct assessment of chondrocytic/osteogenic gene expression and an analysis of the types of collagens (e.g., collagen I versus collagen II) being deposited within the PECMs.

## 5. Conclusions

The results of these studies demonstrated that primary chondrocytes proliferated faster and deposited much higher amounts of extracellular matrix components than chondrocytically induced bMSCs. In addition, the chondrocyte-bMSC cocultures did not significantly enhance the differentiation of bMSCs to a chondrocyte phenotype. Overall, our results suggest that encapsulating primary chondrocytes in polyelectrolyte complex microcapsule modules may be a viable route toward achieving a functional articular cartilage graft.

## Data Availability

Data supporting the results can be requested at hmatthew@wayne.edu.
